# Metabolomics and Antioxidant Activity of Valonea from *Quercus variabilis* Produced in Different Geographical Regions in China

**DOI:** 10.3390/ijms26083599

**Published:** 2025-04-11

**Authors:** Zhenkai Tong, Hao Zhou, Zhiwen Qi, Jianxin Jiang, Wenjun Li, Chengzhang Wang

**Affiliations:** 1 Institute of Chemical Industry of Forest Products, Chinese Academy of Forestry, Nanjing 210042, China; 18036262726@163.com (Z.T.);; 2 College of Materials Science and Technology, Beijing Forestry University, Beijing 100083, China

**Keywords:** *Quercus variabilis*, valonea, bioactive compound, antioxidant capacity, UPLC-ESI-MS/MS, metabolomics

## Abstract

The genus *Quercus* is widely distributed globally and serves as a potential source of phenolic compounds, which are renowned for their potent biological activities. The primary objective of this study was to determine the concentrations of metabolite components and evaluate the relative antioxidant activities of valonea (acorn cups) from *Quercus variabilis* (*Q. variabilis*) of different geographic origins using a UPLC-ESI-MS/MS-based metabolomics approach. A total of 791 metabolite components were identified, with significant variations in their concentrations observed among samples from different geographic locations. Among these, 1-O-galloyl-β-D-glucose was identified as a key active compound. The biosynthesis of galloyl sugars, galactose metabolism, and pathways for starch and sucrose metabolism represent the three pathways that correspond to the differential metabolites, encompassing 23, 11, and 7 metabolites, respectively. The variations in the antioxidant effectiveness of valonea could mainly be linked to the synthesis of galloyl sugars. These findings improve our knowledge of the composition of valonea and offer valuable resources for its extensive utilization and focused development.

## 1. Introduction

The genus *Quercus* (Fagaceae) predominantly comprises forest-dwelling trees and shrubs, forming a vital component of woodland ecosystems worldwide. It stands as one of the most varied plant genera in the Northern hemisphere, with around 450 species [1]. It is distributed extensively across the central, eastern, southwestern, and northern regions of China. The midstream to estuary region of the Yellow River and the basin through which the Yangtze River flows are areas of remarkable diversity for this species. *Quercus*, as a keystone genus, is essential for the structure and functioning of forest ecosystems [2,3,4]. *Quercus variabilis* Blume is a species in the genus *Quercus* of the *Fagaceae* family. Its distinctive features include leaves with spiny awn-shaped serrations along the margins and grayish-white undersides covered with dense stellate hairs. In spring, the tree produces pendulous male catkins as inflorescences emerge, while inconspicuous female flowers develop in the upper leaf axils of the new shoots. The acorns mature in September–October of the following year. The oak nut (seed kernel) is enclosed within a pericarp (acorn shell) that tightly covers approximately 2/3 to 3/4 of the nut. A complete acorn is composed of an oak shell, an oak kernel, and an oak kernel shell. The acorn shell is formed from the involucre of the female flower and is also known as valonea. Valonea, originating from the bracts, represents specialized organs unique to Fagaceae plants and plays a crucial role in safeguarding the seeds. The valonea of *Q. variabilis* are renowned for their abundant tannin content, which notably exceeds that of other tissues [5].

Valonea, characterized by its cup-like shape and hard shell with overlapping lanceolate scales, is a byproduct of the acorn processing industry. Valonea are abundant resources that typically detach from the fruit upon ripening. Despite their abundance, these cup-shaped structures are often underutilized, commonly serving as fuel for energy in industrial operations. Valonea is rich in polyphenolic compounds, with hydrolyzable tannins (HTs) being the primary component. HTs are essential secondary metabolites in plants, providing defense against insects and pathogens. HTs offer various beneficial properties for animals and humans, including bacteriostatic effects, diarrhea prevention, antioxidant activity, and the ability to impart a distinct flavor and astringency to grape wine, earning them the title “the soul of grape wine” [6,7]. Additionally, HTs can be used as a drilling fluid viscosity reducer in the petroleum industry, are employed in the tanning industry, and are utilized as a coagulant in wastewater treatment processes [8,9,10]. Currently, China primarily focuses on the development of products derived from acorn kernels, with a low utilization rate of valonea. Research on the active compounds and biological functions of valonea remains relatively scarce.

Different growing conditions have a significant effect on the quality of plants [11]. Disparities in rainfall patterns, elevation profiles, and diverse environmental factors such as temperature, light intensity, and soil types across the provinces of China may result in differences in the quality of *Q. variabilis* timber. *Q. variabilis*, also known as the Chinese cork oak, is a fast-growing tree species with the ability to withstand drought and thrive in difficult soil environments. Ranging from 22° to 42° N in latitude, the species exhibits a continuous distribution between 24.43° and 40.25° N. *Q. variabilis* is distributed vertically between 50 m and 3000 m [12]. With a distribution range that extends from Liaoning in the north to Guangdong and Yunnan in the south, it is widely distributed across China.

Metabolites are crucial for shaping an organism’s phenotype, contributing to a clearer and more efficient comprehension of biological processes and mechanisms. Metabolomics enables the dissection of metabolic pathways or networks by performing qualitative and quantitative analyses of metabolites. It explores the metabolic underpinnings of macroscopic phenotypes across diverse biological individuals, clarifies how metabolites respond to various physical, chemical, or pathogenic stimuli—such as different diseases or drugs—and carries out safety evaluations of food, pharmaceuticals, and other substances. Metabolomics can further detect and identify metabolites of biological significance with statistically significant differences in biological samples, elucidating the metabolic processes and mechanisms that occur in organisms. The composition of metabolites is typically influenced by ecological factors, which vary across different geographical regions due to diverse climatic conditions, as well as the presence of numerous other species. Li et al. [13] employed a metabolomic comparative strategy to reveal the metabolic profiles of *Lycium barbarum* across various regions. They identified 21 metabolites with potential as biomarkers, demonstrating the efficacy of UHPLC-QTOF-MS/MS in distinguishing the origins of *Lycium barbarum*. Jing et al. [14] performed a study analyzing the metabolic profiles of tobacco leaves with high, medium, and low yields from the Guizhou and Bozhou regions. Their study found that metabolic profiling analysis using GC-MS-targeted metabolomics can successfully distinguish tobacco leaves from various geographical locations and harvests. Such an approach aids in improving our understanding of how metabolites, yield, and geographical location are related. In the same way, the technique of identifying geographical origins using metabolomics has been utilized in a range of disciplines, including soybeans [15], papayas [16], Semen Aesculi [17], sea buckthorn berries [18], and others [19,20,21,22].

Valonea, as an important plant, has been widely recognized for its use in leather making and traditional medicine. However, the impact mechanism of geographical environmental differences on the bioactive components of valonea has not been fully elucidated, which seriously restricts the rational development and utilization of its resources. The aim of this study is to establish a method for analyzing the bioactive components of valonea based on UHPLC-QTOF-MS/MS technology through a broad targeted metabolomics strategy. The main active components of polyphenols, terpenes, alkaloids, etc., in valonea from two typical ecological regions in China (Henan and Guizhou) are qualitatively and quantitatively analyzed to elucidate the impact of geographical environmental differences on the accumulation of active components. This study elucidates the influence of geographical origin on the antioxidant activity of valonea extract and screens for characteristic components with significant antioxidant activity through a three-dimensional evaluation system of DPPH, ABTS+, and FRAP, providing a theoretical basis for the development of natural antioxidants. This study aims to elucidate the molecular mechanisms through which geographical environmental differences influence the accumulation of bioactive compounds in valonea and to screen characteristic ingredients with significant biological activity. By optimizing the development and utilization system of *Q. variabilis* resources with scientific guidance and technological innovation, this research can advance the modernization of the traditional Chinese medicine industry, promote synergistic growth in regionally distinctive economies, and achieve dual improvements in ecological sustainability and economic benefits.

## 2. Results and Discussion

### 2.1. An Overview of the Metabolites

**Figure 1:**
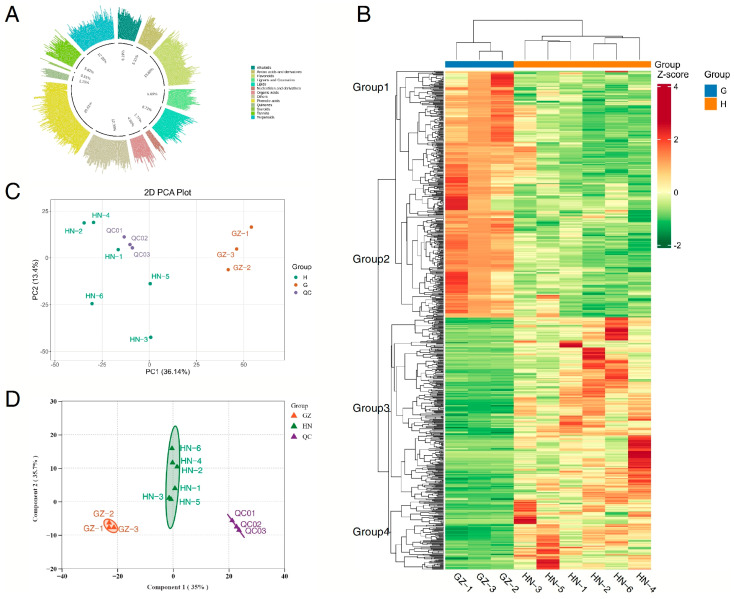
A pie graph, HCA, PCA, and OPLS-DA, showing metabolic traits of valonea originating from the HN and GZ regions. (**A**) The distribution and composition ratios of the main metabolites in the samples. (**B**) HCA was conducted on 791 metabolites present in samples from HN and GZ. (**C**) PCA analysis was performed on 791 metabolites in samples from HN and GZ. (**D**) OPLS-DA analysis was performed on 791 metabolites in samples from HN and GZ.

To better understand the metabolic differences in valonea of *Q. variabilis* across different regions, we employed a comprehensive metabolomic approach using UPLC-MS technology to analyze primary and secondary metabolites in samples collected from Henan and Guizhou. The test results indicate that the number of high-resolution data acquisition signals was 10,972 (positive ion mode) and 11,699 (negative ion mode). A total of 791 metabolites were identified in valonea, with the following composition: 7.28% alkaloids, 7.24% amino acids and derivatives, 15.82% flavonoids, 8.5% lignans and coumarins, 9.11% lipids, 2.44% nucleotides and derivatives, 4.42% organic acids, 14.53% other compounds, 13.04% phenolic acids, 1.33% quinones, 0.34% steroids, 3.32% tannins, and 12.62% terpenoids (Figure 1A). Over 75% of the compounds in the QC samples exhibited a coefficient of variation (CV) < 0.3, indicating highly stable experimental data. The study establishes UPLC-ESI-MS/MS as a precise analytical platform for plant metabolomics, demonstrating exceptional capability in both metabolite quantification and structural elucidation. Notably, valonea emerges as a phytochemically significant species, with its metabolite spectrum dominated by bioactive flavonoids, phenolic derivatives, and terpenoid constituents. While previous studies have mainly focused on tannin metabolites in *Q. variabilis*, the analysis of metabolites in valonea has been limited [23]. To our knowledge, this study represents the first comprehensive investigation that systematically characterizes valonea metabolites within China’s biota, filling the existing knowledge gap on valonea metabolites in the country.

**Figure 2:**
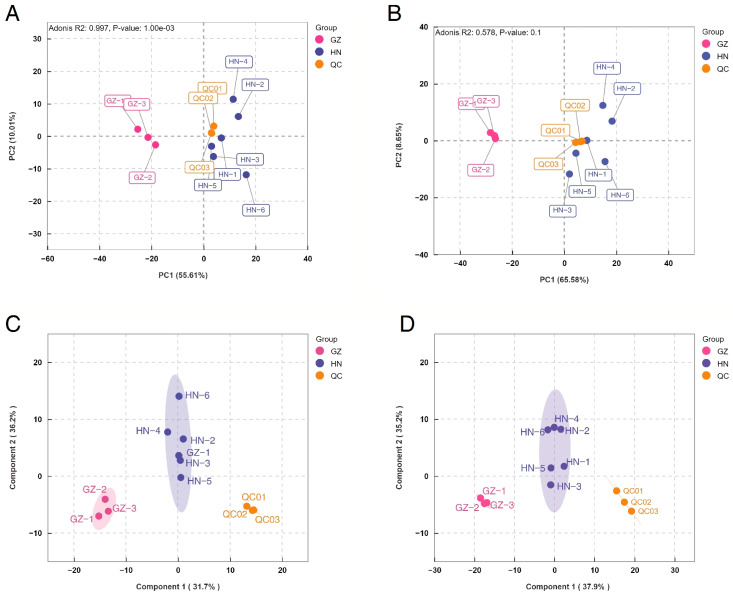
The PCA score plots of valonea samples in the ESI positive ion mode (**A**) and the negative ion mode (**B**). The OPLS-DA score plots of valonea samples in the ESI positive ion mode (**C**) and the negative ion mode (**D**).

PCA was applied to elucidate latent patterns within multivariate datasets through dimensionality reduction. Consequently, differences between groups of valonea from two distinct regions were discernible in the PCA plots (Figure 1C). The first two principal components cumulatively explained 54.79% of the total variance (PC1: 36.14%; PC2: 13.4%). Concurrently, the tight clustering of quality control (QC) samples across both ionization modes (Figure 2A,B) validated strong analytical reproducibility within the experimental workflow. The samples from the GZ group in the PCA score plot demonstrate a strong correlation, while those from the HN group are largely separated. This indicates that PCA was not appropriate for discriminating the metabolites in valonea collected from different regions.

### 2.2. PCA and OPLS-DA of Samples from the Two Regions

In the PCA analysis, the valonea samples from the two regions exhibited poor clustering. To better predict sample classifications and identify potential biomarkers responsible for the differences among the three groups, OPLS-DA was conducted to maximize separation between predefined classes, thereby resolving critical metabolic variations among biological cohorts and enabling the systematic identification of discriminatory metabolites with biomarker potential. Group differences in valonea in two regions can be observed in OPLS-DA plots (Figure 1D). The total variance is explained by PC1 and PC2, with PC1 explaining 35% and PC2 explaining 35.7%, making up 70.7% in total. The OPLS-DA analysis demonstrated a close clustering of the QC samples in both the positive and negative ionization modes (Figure 2C,D), indicating the experiment’s strong reproducibility. Unlike PCA, the valonea samples from the HN and GZ regions exhibited clear and significant separation in the OPLS-DA model. The degree of correlation in the HN group was not as strong as in the GZ group. The HN and GZ groups did not share common metabolites, indicating notable disparities between the two groups and pointing toward a divergence in metabolites between the two regions. This demonstrates inter-group disparities and suggests a trend of separation in metabolites between the two regions, thus highlighting significant differences between the two groups.

### 2.3. HCA of Valonea Samples from the Three Regions

Qualitative variations in metabolite expression levels were employed to conduct hierarchical clustering of sample groups. To visualize the distribution patterns of key metabolites, we generated a heatmap based on HCA. Metabolites grouped together imply that they may participate in closely related reaction steps, with red hues representing higher expression levels and blue hues denoting lower expression levels. The metabolomic heatmap (Figure 1B) delineates origin-specific compositional patterns in valonea, with hierarchical clustering revealing both intergroup metabolic homogeneity and geographically stratified variations in key biomarkers. Furthermore, HCA, based on the Euclidean distance, revealed a clear separation of all samples into two clusters along the horizontal axis, aligning with the results of PCA. Similarly, HCA showed that samples from distinct locations within the same region formed separate clusters, resulting in the classification of four main groups along the vertical axis based on the accumulation of metabolite compounds. Metabolites in groups 1 and 2 were predominantly abundant in the GZ region, whereas metabolites in groups 3 and 4 were more prevalent in the HN region. Multivariate analysis revealed distinct metabolomic signatures that distinguish GZ and HN specimens, with pronounced interregional divergence confirming geographical specificity in secondary metabolite biosynthesis.

**Figure 3:**
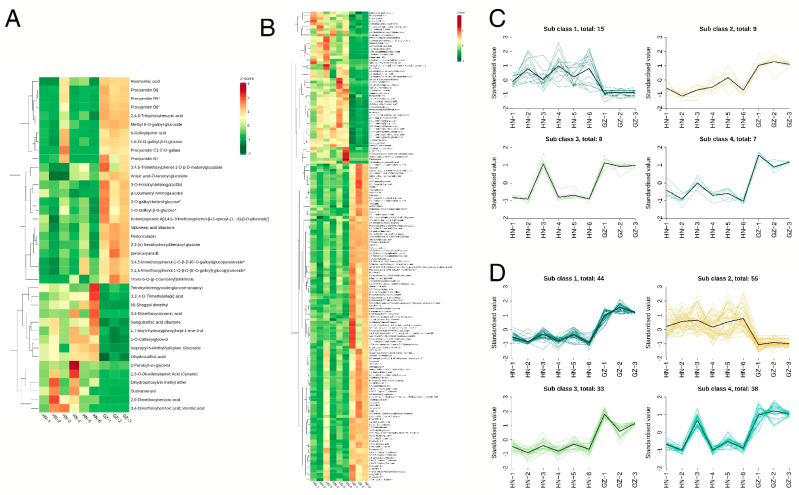
The heatmap illustrates the distribution of identified differential metabolites in the tannin content from the HN and GZ regions under the electrospray ionization (ESI) positive ion mode (**A**) and negative ion mode (**B**). High and low expression levels of the target compounds are represented by red and blue, respectively. Additionally, K-means clustering analysis was performed on the differential metabolites in the ESI positive ion mode (**C**) and negative ion mode (**D**).

Tannins are the important secondary metabolites in valonea. Multivariate analysis of (Figure 3A,B) revealed a dichotomous clustering pattern of tannin profiles, with chemotaxonomic discrimination between samples strictly aligned to geographic provenance across both ionization polarities. Both GZ and HN are distinct branches, in line with the PCA findings, suggesting that the metabolite expression profiles of AH and JZ samples do not show similarity. The selected differential metabolites were subjected to K-means clustering to examine the distinctions of metabolites from different sources. Positive ionization profiling revealed four discrete metabolic clusters through unsupervised hierarchical clustering (Figure 3C). Cluster 1 consisted of 15 metabolites, and cluster 2 contained nine metabolites, with these 24 metabolites exhibiting higher relative expression levels in samples from the GZ origin. Cluster 3 included eight metabolites that were highly expressed in HN-6, while cluster 4 comprised seven metabolites that showed higher relative expression levels in HN-4 but the lowest expression in GZ. In the negative ion mode (Figure 3D), cluster 1 contained 44 metabolites, which demonstrated relatively high expression levels in HN. Cluster 2 contained 55 metabolites, cluster 3 contained 33 metabolites, and cluster 4 contained 38 metabolites; a total of 126 metabolites showed high expression levels in samples of GZ origin.

### 2.4. Identification and Analysis of Candidate Differential Metabolites

**Figure 4:**
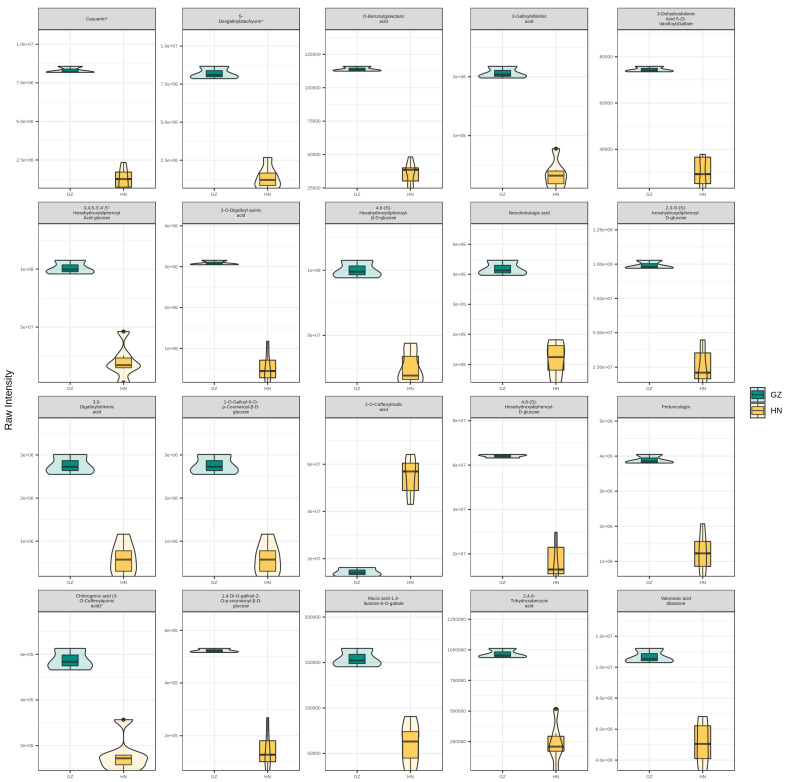
A boxplot displaying the normalized peak areas of differential metabolites in valonea samples from two geographical origins, Guizhou and Henan.

By employing univariate statistical analysis methods, including hypothesis testing and fold change, combined with multivariate statistical analysis approaches such as OPLS-DA, a thorough analysis was conducted from multiple perspectives based on the data characteristics. Using the OPLS-DA model with biological replicates ≥ 3, we applied variable importance in projection (VIP) to identify and investigate metabolites that displayed differences between the two regions. The stringent screening criteria required metabolites to have a VIP of ≥1, a fold-change ≥ 2 or ≤0.5, and a *p*-value of <0.05 to be considered potential marker compounds. The relative concentrations of differential metabolites in the valonea samples are illustrated in corresponding violin plots, highlighting only the top 20 differential metabolites with the highest VIP values, given that the total number of differential metabolites exceeded 20. Between the GZ and HN groups, 20 tannin constituents displayed notable differences. The data from boxplots demonstrate a significant upregulation in the concentration of all tannin markers in the GZ group, except for caffeoylmalic acid. Glucose was most abundant in the GZ group, with casuarinin and valoneaic acid dilactone following closely behind (Figure 4).

### 2.5. KEGG Annotation

**Figure 5:**
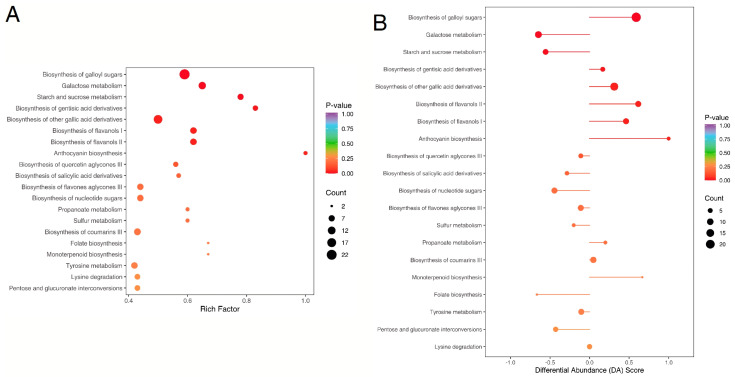
(**A**) Multivariate KEGG enrichment analysis of differential metabolites visualized through parametric bubble mapping: horizontal coordinates integrate multidimensional analytical metrics, with the bubble diameter scaled to the pathway impact magnitude. Chromatic intensity correlates with enrichment significance (warmer hues denote stronger pathway enrichment). (**B**) Stratified profiling of differential abundance (DA) scores across metabolic networks, demonstrating pathway-specific regulation patterns in comparative groups.

KEGG-based pathway annotation of 791 differential metabolites successfully mapped 698 compounds to 99 canonical metabolic pathways, demonstrating comprehensive coverage (88.2%) across primary and secondary metabolism functional modules, with only two pathways attributed to genetic or environmental factors. This indicates that the majority of differential metabolites are associated with valonea metabolism. As the *p*-value approaches 0, the enrichment becomes more noticeable. The biosynthesis of galloyl sugars, galactose metabolism, and pathways for starch and sucrose metabolism represent the three pathways corresponding to the differential metabolites in the HN and GZ groups, encompassing 23, 11, and 7 metabolites, respectively (Figure 5). A total of 24 metabolites showed an increase, whereas 17 metabolites showed a decrease. The Differential Abundance Score (DA Score) was used to characterize the overall trends of metabolite changes in metabolic pathways and holds significant reference value. The directional tendency metric (1 = pathway activation, −1 = metabolic suppression) is visualized through horizontal bar magnitude (|DA score|) and elliptical topology, where spatial dimensions encode the effect intensity and differential metabolite density within the biochemical pathways. If the circle extends to the left of the intermediate axis and is far away, it indicates that the expression of this pathway is generally downregulated. On the contrary, if the circle extends to the left of the intermediate axis and is far away, it indicates an overall upward trend, and the larger the circle, the more metabolites it contains. The color gradient of graphical elements (red to purple) reflects statistical significance levels, with warmer hues indicating lower *p*-values. Among the three significantly altered metabolic pathways detected in this analysis, galloyl sugar biosynthesis exhibited increased activity. Conversely, both galactose utilization and carbohydrate storage mechanisms involving starch/sucrose conversion demonstrated reduced metabolic flux. Consistent with previous research, tannins are the most important secondary metabolites in valonea, comprising gallic acid, ellagic acid, procyanidin, cinnamtannin, and catechin [5,23]. Their antioxidant and antibiotic properties have positioned them as longstanding “natural antibiotics” for livestock and poultry, with a history dating back centuries [24,25,26,27]. The consumption of anthocyanins has been linked to a potential decrease in the risk of cardiovascular diseases, as suggested by various epidemiological studies and human intervention trials highlighting their anti-thrombotic effects [28,29]. Gallic acid exhibited anti-urolithiatic effects by inhibiting the formation of urolithiasis crystals, thereby enhancing renal health [30].

Studies have revealed that the biosynthesis of HTs begins with the formation of 1-O-galloyl-β-D-glucose via the condensation of uridine diphosphate-glucose and gallic acid, representing a crucial step in HT biosynthesis [31]. In the present research, during the biosynthesis of galloyl sugars, the concentrations of metabolites such as 1-O-galloyl-β-D-glucose, 1,6-di-O-galloyl-β-D-glucose, 1,2,6-di-O-galloyl-β-D-glucose, and 1,4,6-di-O-galloyl-β-D-glucose were detected in both the GZ and HN samples. Nevertheless, the levels of these metabolites were notably elevated in the GZ and HN3 samples in comparison to the other HN samples. Research has shown that, apart from 1-O-galloyl-β-D-glucose, the levels of other compounds in the HT biosynthesis pathway did not exhibit a consistent pattern with the total HT content. The correlation coefficients of these compounds with the total HTs were lower than those of 1-O-galloyl-β-D-glucose. At the same time, research has also identified 1-O-galloyl-β-D-glucose as a marker metabolite for HTs in various species of Asian oaks [23]. The compound β-glucoside has shown great potential as a candidate drug for the treatment of diabetes-related problems. A clinical investigation demonstrated comparable efficacy between a 10% β-glucoside-enriched Emblica officinalis extract (EOE) and metformin in managing diabetes-induced dyslipidemia. The results showed that taking EOE-2 g once a day demonstrated comparable efficacy to the drug preparation metformin in producing anti-diabetes and anti-dyslipidemia effects [32]. The study found that β-glucoside is the main component of Emblica officinalis, which has a selective and relatively effective inhibitory effect on aldo keto reductases (AKRs) in vitro, and β-glucagon effectively inhibits sorbitol in the lens excised from transgenic mice in a vitro organ culture model, providing a new method for the treatment of cataracts and other diabetes complications [33]. There are also research results that indicate that using β-glucoside on retinal cells to alleviate intraocular pressure may be a promising treatment for controlling glaucoma [34]. The protective role of 1-O-galloyl-β-D-glucose in safeguarding humans against diabetes and related complications, such as retinopathy, glaucoma, inflammation, liver damage, and UV-induced skin injuries, elucidates the traditional medicinal application of oak [35]. Some studies focused on the analysis of certain drugs for the treatment of diabetes, such as the glucagon-like peptide 1 (GLP-1) agonist, the glucose-dependent insulin-stimulating polypeptide (GIP) agonist, and the GLP 1/GIP dual agonist, which have many beneficial effects in improving the prognosis of disease, and some new molecules, such as quinone pimaric acid derivatives, can be used to inhibit some enzymes involved in glucose metabolism [36]. Current pharmacological investigations remain constrained by insufficient translational studies spanning preclinical models to clinical observations, impeding the systematic characterization of these therapeutic agents’ polypharmacological networks and multifunctional signaling cascades. The same applies to β-glucoside discovered in this study, which currently lacks an understanding of its effects and efficacy and therefore requires further research.

**Figure 6:**
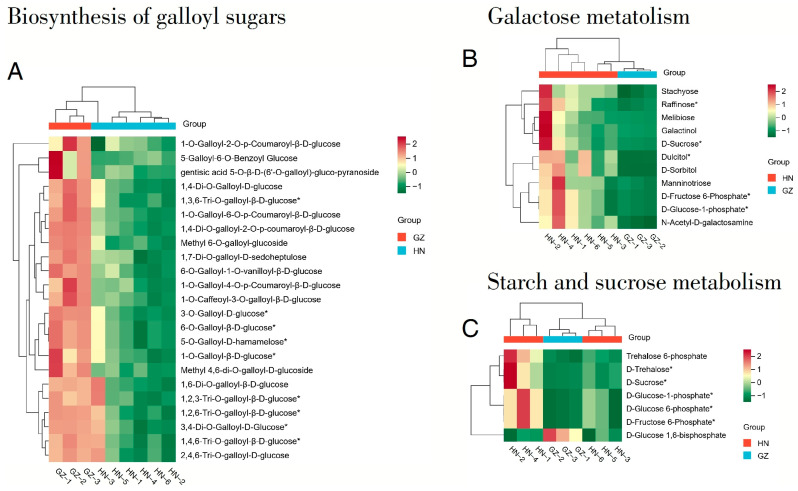
A heatmap illustrating the levels of metabolites associated with significant differential metabolic pathways between HN and GZ. (**A**) The biosynthetic pathway of galloylated sugars in HN and GZ samples. (**B**) Galactose Metabolic Pathway in HN and GZ Samples. (**C**) Starch and Sucrose Metabolic Pathway in HN and GZ Samples. The compounds marked with an asterisk (*) are isomers.

A total of 11 metabolites are involved in galactose metabolism (including N-acetyl-D-galactosamine, mannotriose, dulcitol, D-sorbitol, galactitol, melibiose, D-fructose, D-sucrose, 6-phosphate, D-glucose-1-phosphate, raffinose, and stachyose), while seven metabolites participate in starch and sucrose metabolism (such as D-trehalose, trehalose 6-phosphate, D-glucose 6-phosphate, D-glucose 1,6-bisphosphate, D-sucrose, D-fructose-6-phosphate, and D-sugar-1-phosphate; Figure 6). Additionally, plant secondary metabolites are vital for boosting plant survival and fostering ecological relationships with other organisms. Through the synthesis of compounds like alkaloids, terpenoids, phenolics, and tannins, they enable plants to combat pathogens, herbivores, and environmental challenges, thereby strengthening their resilience. Moreover, these metabolites play a key role in symbiotic interactions, such as facilitating nitrogen fixation by rhizobia, which improves nutrient acquisition. In ecosystems, they contribute to maintaining balance by shaping food web dynamics and nutrient cycles. As a result, secondary metabolites are indispensable for plant adaptation and the overall stability and functionality of ecosystems [37]. Hence, the enrichment of the mentioned metabolites in valonea may suggest their favorable physiological roles and capacity to withstand environmental challenges.

### 2.6. In Vitro Antioxidant Activities and Total Tannins Analysis

**Figure 7:**
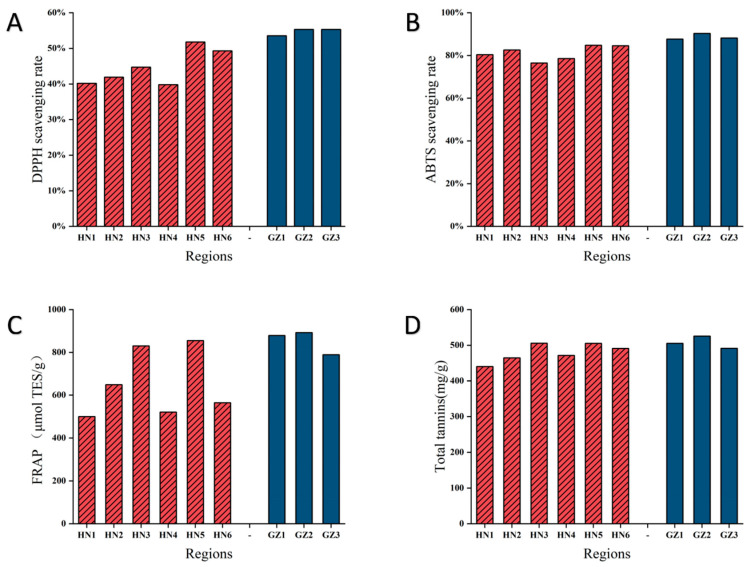
In vitro antioxidant activity and total tannin content analysis of valonea samples from the HN and GZ regions. (**A**) The DPPH radical scavenging capacity. (**B**) The ABTS radical scavenging activity. (**C**) The ferrous ion chelating activity. (**D**) The total tannin content. The cyan bars represent samples from the HN region, while the blue bars represent samples from the GZ region.

It has been previously demonstrated that the antioxidant capacity of valonea is impacted by the tannin levels within them. We evaluated the in vitro antioxidant activity of valonea from the HN and GZ regions to analyze the correlation between antioxidant capacity and changes in tannin concentration. This study included measurements of antioxidant capacities such as DPPH and ABTS radical scavenging activities, as well as FRAP assays, with the results shown in Figure 7. Notably, a significant positive correlation was observed between the tannin concentration and the antioxidant activities measured by ABTS and DPPH assays in the GZ group. This indicates that higher levels of tannins in the GZ group were associated with increased antioxidant capacity, as demonstrated by the ABTS and DPPH methods. Tannins, known for their potent antioxidant properties, likely contribute to the enhanced ability to neutralize free radicals and reduce oxidative stress. These findings suggest that tannins play a key role in the antioxidant potential of the GZ group, highlighting their importance in combating oxidative damage and supporting overall plant defense mechanisms. The majority of tannin compounds exhibit significant potential as antioxidants. For instance, gallic acid, ellagic acid, procyanidin, cinnamtannin, and catechin have all shown effective free radical scavenging abilities and FRAP. Antioxidants are naturally present in many beverages, fruits, and vegetables, and they have multiple benefits in preventing and treating diseases. Studies have evaluated the value of antioxidants in improving drug and chemical-induced toxicity, reducing oxidative stress, and preventing aging, metabolism, and degenerative diseases [38,39]. Galván et al. used FRAP and ABTS to determine the total antioxidant activity of *Q. cacifera* seeds, the results showed that ABTS and FRAP measurements were directly related to the content of total polyphenols, total phenols, and total flavonoids, consistent with the findings of this study [40]. Contrary to findings in related *Quercus* species, interspecific comparisons revealed a decoupling between the total polyphenolic content and antioxidative efficacy in acorn extracts when assessed via DPPH/ABTS assays [41]. Álvarez et al. evaluated the use of *Quercus* extract in vitro and computer simulations to assist in the treatment of depression and anxiety and analyzed its antioxidant components as monoamine oxidase inhibitors, the results showed that some glycosylated derivatives, such as gallic acid, chlorogenic acid, quinic acid and shikimic acid, quercetin glucuronide, tannic acid, and tannic acid methyl ether, could effectively inhibit monoamine oxidase A, thus having the potential to treat depression and anxiety [42]. In a study focusing on *Q. variabilis* acorn kernels, Du et al. identified five bioactive polyphenols—gallic acid, quercetin, azelaic acid, ellagic acid, and ferulic acid—that effectively attenuated Alzheimer’s disease pathogenesis in APP/PS1 transgenic mouse models, establishing critical pharmacological insights for therapeutic development [43].

## 3. Materials and Methods

### 3.1. Plant Materials, Chemicals, and Reagents

In October 2024, valonea samples were collected from six *Q. variabilis* cultivation counties in Henan Province, China, including Xixia (HN-1), Nanzhao (HN-2), Xichuan (HN-3), Luanchuan (HN-4), Ruyang (HN-5), and Zhenping (HN-6), and three *Q. variabilis* cultivation counties in Guizhou Province, including Wangmo (GZ-1), Luodian (GZ-2), and Changshun (GZ-3). Table 1 displays the fundamental and geographical data pertaining to the valonea samples. The sample collection method was as follows: for each county, five healthy 8–10-year-old *Q. variabilis* trees cultivated on completely flat land with slopes of 5 to 15 degrees were selected. Approximately 50 mature acorns were collected per tree by harvesting hardened valonea from the periphery and the top of the tree toward the center. Acorns from the five trees in each county were uniformly mixed. The acorns and valonea were separated, sprayed with alcohol, stored in low-temperature containers, and transported to the laboratory for preservation at −80 °C. All trees were identified as *Q. variabilis* by professors at our institute and cross-referenced with records in Flora of China. Complete fruit samples from each tree were compared with herbarium specimens at the Institute of Botany, Chinese Academy of Sciences, to confirm species identity.

Ultrapure water (18.2 MΩ·cm, CAS 7732-18-5) was obtained from Millipore (Burlington, MA, USA). Methanol (HPLC grade, CAS 67-56-1) and L-ascorbic acid (≥99%, CAS 50-81-7) were purchased from Sigma-Aldrich (St. Louis, MO, USA). Formic acid (≥98%, CAS 64-18-6) and sodium carbonate (≥99.5%, CAS 497-19-8) were sourced from Merck (Darmstadt, Hesse, Germany). Acetonitrile (HPLC grade, CAS 75-05-8) was supplied by Thermo Fisher Scientific (Waltham, MA, USA). Potassium persulfate (analytical grade, CAS 7727-21-1) and iron(III) chloride (99%, CAS 7705-08-0) were acquired from Aladdin (Shanghai, China) and Sinopharm Chemical Reagent Co. (Shanghai, China), respectively. Acetic acid (≥99.5%, CAS 64-19-7) was provided by J&K Scientific (Beijing, China). Folin-Ciocalteu reagent (2 mol/L aqueous solution, Product No. 476650-100ML) was obtained from Sigma-Aldrich (St. Louis, MO, USA).

### 3.2. Sample Processing and Quality Control

Nine frozen samples from nine counties across two provinces in China were removed from the freezer. Twenty valonea of uniform size were selected from each sample and placed in a vacuum freeze-dryer (Scientz-100F, Scientz Biotechnology Co., Ltd., Hangzhou, China) for 60 h to remove moisture. Processed samples were pulverized using a MM 400 ball mill (Retsch, Haan, Germany) following dehydration, with two 3 mm glass grinding media ensuring particle size reduction. Grinding conditions were set to 30 Hz of power, a 1.5 min duration, and a temperature of −10 °C. The ground samples were sieved through a 40-mesh sieve. Using an electronic balance (MS105DM, Mettoltoldo Instruments Ltd., Zurich, Switzerland), 50 ± 5 mg of each powdered sample was accurately weighed into 2 mL centrifuge tubes. A 1200 μL extraction solution (methanol:water = 7:3, *v*/*v*, pre-cooled to −20 °C) was added to each tube. The homogenate was vortexed (VORTEX-5, Kyllin-Bell, Haimen, China) for 30 s, allowed to rest for 6 min, and vortexed again, repeating this cycle three times. Following vortexing, samples were incubated on ice for 30 min and centrifuged (12,000 rpm, 15 min, 4 °C). The resulting supernatant was filtered through 0.22 μm organic microporous membranes (Thermo Scientific, Waltham, MA, USA). The filtrate was stored in 2 mL injection vials for subsequent analysis. For quality control (QC), pooled samples (QC1, QC2, and QC3) were prepared during grinding by randomly mixing material from at least three of the nine solid samples. These served as three technical replicates. To ensure uniform processing conditions, all samples were extracted, processed simultaneously, and analyzed sequentially. The QC samples were inserted after every six experimental samples during instrument analysis to validate methodological reliability and monitor instrument stability.

### 3.3. UPLC-ESI-MS/MS Analysis

The valonea extracts were analyzed via an Ultra Performance Liquid Chromatography-Electrospray Ionization Tandem Mass Spectrometry (UPLC-ESI-MS/MS) system (Waters, Milford, MA, USA). Chromatographic separation was carried out on an Agilent SB-C18 column (1.8 µm, 2.1 × 100 mm; Santa Clara, CA, USA) with a mobile phase of (A) 0.1% formic acid in water and (B) 0.1% formic acid in acetonitrile. The method operated at a flow rate of 0.35 mL/min, with a 2 μL injection volume and a column temperature of 40 °C. A gradient elution program was applied as follows: 0–9 min (5–95% B), 9–10.01 min (95% B), 10.01–11.01 min (95–5% B), and 11.01–14.01 min (5% B). The effluent was then analyzed by an Electrospray Ionization Quadrupole Linear Ion Trap Tandem Mass Spectrometer (ESI-Q TRAP-MS/MS).

Mass spectrometric analysis utilized an electrospray ionization (ESI) source at 500 °C. Gas parameters included 50 psi for both the nebulizer (GS1) and auxiliary heating (GS2) gases, with a curtain gas (CUR) pressure of 25 psi. The ion spray voltage (IS) was configured to 5500 V (positive mode) or −4500 V (negative mode). The declustering voltage (DP) and collision energy (CE) were adjusted to 60 V and 10 eV, respectively, with polarity-dependent sign adjustments (±). A triple quadrupole mass spectrometer (QQQ) operated in multiple reaction monitoring (MRM) mode, employing nitrogen as the collision gas at medium intensity. For each metabolite-specific MRM transition, DP and CE parameters were further optimized.

Time-of-flight mass spectrometry (TOF-MS) analysis employed precursor and product ion scan ranges of 100–1250 Da and 50–1250 Da, respectively, with accumulation times of 150 ms and 30 ms. Dynamic background subtraction was activated during data acquisition. The CE was set to ±30 V (positive/negative mode), the collision energy spread (CES) to 10 V, and the mass tolerance to 50 ppm. Each valonea extract was analyzed in three independent technical replicates, and the results were averaged to ensure data reliability. This method was consistently applied to all samples.

### 3.4. Metabolite Profiling Analysis

The raw data were processed using MassLynx V4.1 (Waters, Milford, MA, USA) via its information-dependent acquisition (IDA) functionality. To address retention time (RT) drift, a dynamic time warping (DTW) algorithm was employed to achieve cross-sample peak alignment by integrating m/z values, RT, and peak shape characteristics (peak width, symmetry). Low-reproducibility peaks (detected in less than 20% of the samples) were filtered out. Missing values were imputed using either half of the detection limit, the minimum observed value in the sample, or statistical models. Isotopic peaks and adducts were merged through mass difference matching, abundance pattern verification, and correlation with precursor ion MS/MS spectra. Metabolite structural annotation was performed using the MetWare database (MWDB), incorporating precursor ion mass accuracy (MS error ≤ 20 ppm), intelligent MS/MS spectral matching (fragment ions, isotopic distribution), and RT consistency (deviation ≤ 0.2 min). The confidence levels for metabolite identification in this experiment strictly adhered to the internationally recognized four-tier classification system of the Metabolomics Standards Initiative (MSI), combined with orthogonal data validation methods: Level 1 (identified metabolites) required the secondary mass spectra (all fragment ions) of the sample to match laboratory-internal authentic chemical standard database spectra with a score ≥ 0.7, along with retention time (RT) consistency with the standard, and further validation through isotopic distribution similarity or derivatization experiments; Level 2 (putatively annotated compounds) were annotated based on secondary mass spectral matching scores of 0.5–0.7 and spectral feature similarity to databases, but lacked support from laboratory-specific standards; Level 3 (putatively characterized compound classes) relied on matching parameters such as precursor ion (Q1), product ion (Q3), CE, DP, and physicochemical property characteristics of specific chemical classes; and Level 4 (unknown compounds) represented unidentifiable or unclassified metabolites (see Table S1). The confidence level assignments fully aligned with international standards: Level 1 required at least two orthogonal datasets (e.g., RT and secondary spectra) matched to laboratory-analyzed authentic standards, while Levels 2/3 relied on singular or non-orthogonal data. All metabolites were explicitly labeled with their identification levels to enhance traceability [44,45]. To ensure accurate cross-sample quantification, this study employed a dynamic time warping (DTW) algorithm to integrate the precise mass-to-charge ratio (*m*/*z*), retention time (RT), and chromatographic peak characteristics (peak width, symmetry) for nonlinear alignment of chromatographic peaks. This alignment was optimized through multidimensional parameter matching (*m*/*z* deviation ≤ 0.01 Da, RT window ± 0.5 min, peak shape similarity > 80%). Subsequently, low-reproducibility metabolite signals detected in fewer than 20% of samples were filtered out, and missing values were uniformly imputed using half of the metabolite-specific detection limit (LOD/2), calculated based on the signal-to-noise ratio threshold (S/N = 3). Isotope peaks, fragment peaks, and adducts were summarized using mass-to-charge ratio difference and abundance pattern recognition, fixed mass difference matching, and MS/MS spectral correlation of parent ions. Metabolite composition analysis revealed significant inter-sample differences in metabolite classes and proportions. Method reproducibility was validated using QC samples, with metrics such as the peak area coefficient of variation (CV) and RT stability ensuring experimental reliability.

### 3.5. In Vitro Antioxidant Activity Assay

All experimental kits were sourced from Shanghai Jining Biotechnology Co., Ltd. (Shanghai, China). The clearance rate of 2,2′-azino-bis(3-ethylbenzothiazoline-6-sulfonate) diammonium (ABTS+) was determined using standardized protocols [46]: ABTS and potassium persulfate were weighed and a solution of each was prepared in purified water; 250 μL of each solution was mixed, incubated in the dark for 6 h, and diluted 35 times with PBS to obtain the ABTS stock solution. Next, a volume of 10 μL of the valonea extract sample solution was withdrawn and 200 μL of the ABTS stock solution was added to form the sample group; 10 μL of pure water and 200 μL of PBS working solution were mixed in sequence as the sample control group; 10 μL of the (L-ascorbic acid/vitamin C, VC) solution and 200 μL of ABTS solution were mixed as a positive control; 10 μL of PBS and 200 μL of ABTS stock solution were mixed as blank groups. Three parallel samples were set up for each group, and the absorbance values of all sample groups were measured at 540 nm. The calculation formula for the ABTS clearance rate was as follows:

ABTS clearance rate = [1 − (A sample − A control)/A blank] × 100%

The determination of the clearance rate for 2,2-biphenyl-1-picrylhydrazyl (DPPH) was performed as follows: DPPH was weighed and placed in a volumetric flask; methanol was added to prepare the DPPH storage solution with a concentration of 1 mmol/L, and was stored in the dark for future use; 4 mg/mL of different extract solutions was prepared and diluted 1:1 to 0.125 mg/mL for later use. Next, 20 μL of the valonea extract sample solution was withdrawn and 0.6 mL of the DPPH storage solution was added, using pure water as a blank control; the solutions were mixed and incubated at room temperature in the dark for 30 min; next, they were centrifuged at 5000 r/min for 2 min; the DPPH storage solution was replaced with methanol solution in the control group; finally, 0.2 mL was withdrawn to prepare 3 parallel wells and the absorbance was measured at 540 nm.

The formula for calculating the DPPH clearance rate was as follows:

DPPH clearance rate = [1 − (A sample − A control)/A blank] × 100%

The ferric ion reducing antioxidant power (FRAP) assay was performed using a TAOCF-W96-N (1620) kit (Jining Industrial, Shanghai, China) following the manufacturer’s guidelines: 10 μL of the valonea extract sample solution was withdrawn and mixed with 20 mmol/L of the FeCl_3_ solution and 0.1 mol/L of the acetic acid buffer solution (pH 3.6) in a ratio of 1:1:10; fresh FRAP reagent was prepared by mixing with the sample solution and was incubated in the dark for 30 min. The absorbance was measured at 593 nm.

### 3.6. Determination of Total Tannins Content

The total tannins were quantified using the Folin–Ciocalteu method, adhering to the protocol described by Yang et al. [47], with slight modifications. A standard curve was prepared using tannic acid (Sigma-Aldrich): 10.0 mg was dissolved in 100 mL distilled water to generate a 0.1 mg/mL stock solution. Aliquots of 0, 0.1, 0.2, 0.4, 0.6, and 0.8 mL from the stock solution were transferred to 10 mL volumetric flasks, adjusted to 1 mL with distilled water, mixed with 0.5 mL of the Folin–Ciocalteu reagent (1 mol/L), and incubated for 5 min. Next, 4 mL of 7.5% (*w*/*v*) Na_2_CO_3_ was added, diluted to volume, and incubated for 30 min at room temperature (light-protected). Absorbance at 765 nm was measured against an ultrapure water blank, with triplicate replicates averaged per concentration.

For the samples, 0.5 mL of the methanol extract (or 1 mL ultrapure water for the blank group) was substituted for the standard solution, following the same procedure. The total tannin content was calculated as follows:

Total Tannins (mg/g) = (C × V × 2 × 10^−3^)/M

C-Tannic acid concentration (mg/L) derived from the standard curve; V-Volume of the test solution (mL); M-Sample mass (mg).

### 3.7. Statistical Analysis

This study employed multivariate analytical methods to process metabolomics data. For unsupervised analysis, Principal Component Analysis (PCA) was implemented using the R platform, and Hierarchical Cluster Analysis (HCA) was conducted on metabolite data from all samples, with clustering results visualized as a dendrogram heatmap. Key variable screening utilized Orthogonal Partial Least Squares-Discriminant Analysis (OPLS-DA), and variable importance in projection (VIP) scores were extracted via the MetaboAnalystR 4.0 package. Metabolite annotation was performed using the Kyoto Encyclopedia of Genes and Genomes (KEGG) compound database, followed by pathway enrichment analysis mapped through the KEGG Pathway database. During data analysis, the R package ComplexHeatmap 2.16.0 was applied for heatmap visualization, SPSS 22.0 (IBM; Armonk, NY, USA) was used for statistical calculations, and graphical visualization was achieved using the OriginPro 2021b software (OriginLab Corporation, Northampton, MA, USA).

## 4. Conclusions

For a considerable period, oaks have been pivotal model plants in the study of evolution and interspecific hybridization within ecology. Using UPLC-ESI-MS/MS technology, a comprehensive targeted metabolomics analysis was conducted on the metabolites of *Quercus* plants from the Henan and Guizhou regions, followed by systematic identification and comparison. The experimental findings revealed variations in metabolite levels between Chinese valonea sourced from the two regions. Ecological factors typically influence the composition of metabolites, a phenomenon particularly evident in Henan. 1-O-galloyl-β-D-glucose was identified as a key active compound, significantly enhancing our understanding of the primary active ingredient in acorn fruits. These findings can assist researchers in selectively targeting regions to meet breeding or natural product extraction requirements.

## Figures and Tables

**Table 1:** Specific details regarding valonea samples.

Province	Area	Longitude (°E)	Latitude (°N)	Size (cm)	Degree of Ripeness	Climatic Conditions	The Color of the Acorn	Range of the Valonea Package
Henan	HN-1	111.48	33.28	3–4	fully mature	Warm Temperate Continental Monsoon Climate	dark red	2/3
	HN-2	112.49	33.29	3–4	fully mature	Warm Temperate Continental Monsoon Climate	dark brown	2/3
	HN-3	111.49	33.14	3–4	fully mature	Warm Temperate Continental Monsoon Climate	dark brown	2/3
	HN-4	111.62	33.78	2–3	fully mature	Warm Temperate Continental Monsoon Climate	dark red	2/3
	HN-5	112.47	34.15	2–3	fully mature	Warm Temperate Continental Monsoon Climate	dark brown	2/3
	HN-6	112.14	33.02	3–4	fully mature	Warm Temperate Continental Monsoon Climate	dark brown	2/3
Guizhou	GZ-1	106.09	25.17	2–3	fully mature	Subtropical Humid Monsoon Climate	dark red	3/4
	GZ-2	106.75	25.43	2–3	fully mature	Subtropical Humid Monsoon Climate	dark brown	3/4
	GZ-3	106.45	26.03	2–3	fully mature	Subtropical Humid Monsoon Climate	dark red	3/4

## Data Availability

The original contributions presented in the study are included in the article/Supplementary Materials. Further inquiries can be directed to the corresponding author.

## Supplementary Materials

The following supporting information can be downloaded at https://www.mdpi.com/article/10.3390/ijms26083599/s1.
